# Imperatoxin A, a Cell-Penetrating Peptide from Scorpion Venom, as a Probe of Ca^2+^-Release Channels/Ryanodine Receptors

**DOI:** 10.3390/ph3041093

**Published:** 2010-04-13

**Authors:** Georgina B. Gurrola, E. Michelle Capes, Fernando Z. Zamudio, Lourival D. Possani, Héctor H. Valdivia

**Affiliations:** 1 Departamento de Medicina Molecular y Bioprocesos, Instituto de Biotecnología, Universidad Nacional Autónoma de México, Cuernavaca, Morelos 62271,Mexico; E-Mails: georgina@ibt.unam.mx (G.B.G.); possani@ibt.unam.mx (L.D.P.); zam@ibt.unam.mx (F.Z.Z.); 2 Department of Physiology, University of Wisconsin Medical School, Madison, WI 53706, USA; E-Mail: capes@physiology.wisc.edu (E.M.C.)

**Keywords:** imperatoxin, ryanodine receptors, cell-penetrating peptide, calcin, drug delivery

## Abstract

Scorpion venoms are rich in ion channel-modifying peptides, which have proven to be invaluable probes of ion channel structure-function relationship. We previously isolated imperatoxin A (IpTxa), a 3.7 kDa peptide activator of Ca^2+^-release channels/ryanodine receptors (RyRs) [1,2,3] and founding member of the calcin family of scorpion peptides. IpTxa folds into a compact, mostly hydrophobic molecule with a cluster of positively-charged, basic residues polarized on one side of the molecule that possibly interacts with the phospholipids of cell membranes. To investigate whether IpTxa permeates external cellular membranes and targets RyRs *in vivo*, we perfused IpTxa on intact cardiomyocytes while recording field-stimulated intracellular Ca^2+^ transients. To further investigate the cell-penetrating capabilities of the toxin, we prepared thiolated, fluorescent derivatives of IpTxa. Biological activity and spectroscopic properties indicate that these derivatives retain high affinity for RyRs and are only 5- to 10-fold less active than native IpTxa. Our results demonstrate that IpTxa is capable of crossing cell membranes to alter the release of Ca^2+^ *in vivo*, and has the capacity to carry a large, membrane-impermeable cargo across the plasma membrane, a finding with exciting implications for novel drug delivery.

## 1. Introduction

The majority of Ca^2+^ required for muscle contraction during excitation-contraction coupling in cardiac and skeletal muscle is released into the cytoplasm by ryanodine receptors (RyRs), the Ca^2+^ release channels of the sarcoplasmic reticulum [4]. RyRs are found in a diverse array of cell types, including neurons, exocrine cells, smooth muscle cells, epithelial cells, lymphocytes, and sea urchin eggs. In all of these cells, RyRs function in the regulation of the intracellular free Ca^2+^ concentration ([Ca^2+^]_i_), which, when elevated, triggers a tissue-specific cascade of events that result in muscle contraction, hormone secretion, lymphocyte activation, fertilization, and many other physiological processes [2]. Ryanodine, an alkaloid from the South American plant *Ryania speciosa*, is the major probe used for structural and functional studies of RyRs because of its selectivity and high affinity for these channels. The alkaloid is extremely useful as a probe for pharmacological profiling of RyRs, but some of its properties, such as slow association and dissociation rates, and concentration-dependent behavior as both agonist and antagonist, are undesirable features that limit its usefulness in experiments with intact cells [1,2], a necessity for unraveling complex Ca^2+^ signaling events in physiologically relevant conditions. 

The search for a more useful probe of RyRs uncovered imperatoxin A (IpTxa), a ;3.7 kDa peptide isolated from the venom of the African scorpion *Pandinus imperator* which, unlike ryanodine, activates RyRs in a reversible manner, making it an invaluable tool for investigating the molecular events underlying excitation-contraction coupling in intact cells. The toxin is a basic peptide with a net charge of +8, with almost all positive charges of the basic residues oriented on one pole of its globular structure. IpTxa forms an inhibitor cystine knot (ICK) fold, a structural motif that is evolutionarily conserved among a wide array of polypeptides, but, most importantly, among many calcium channel-modifying toxins [5]. Indeed, IpTxa has been shown to enhance binding of [^3^H]ryanodine to sarcoplasmic reticulum vesicles, and to induce subconducting states in single RyR channels reconstituted into lipid bilayers [6]. IpTxa’s RyR-activating capabilities piqued our curiosity because a scorpion toxin that is targeted to an *intracellular* ion channel seems counterintuitive. However, Maurocalcin (MCa), a RyR-activating toxin isolated from *Scorpio maurus palmatus* based on sequence similarity to IpTxa, has been shown to translocate across cell membranes [7]. MCa shows similarities with known cell-penetrating peptides including penetratin, derived from the Antennapaedia homeodomain, and the Tat protein transduction domain, derived from HIV and equine infectious anemia. MCa, in addition to being amphipathic in its globular structure, was noted to possess in its sequence a stretch of positively-charged residues resembling a protein translocation domain. 

The high degree of sequence identity between MCa and IpTxa and the known biological activity of IpTxa on RyRs compelled us to investigate whether IpTxa is capable of translocating across the cell membranes of intact cardiomyocytes. In the current study, we used confocal microscopy to perform Ca^2+^ release studies with native toxin, and a fluorophore-marked IpTxa to investigate translocation across membranes. We describe a method for the preparation of IpTxa derivatives containing fluorescent probes, and demonstrate that the modified toxin is biologically active and retains high binding affinity for RyR. Its activity is not appreciably altered, indicating preservation of native structure, which makes this compound useful for the study of the structure and dynamics of RyRs. This presents an attractive alternative to radioisotopes because fluorescent probes are not subject to regulation and do not require special handling or disposal. In addition, fluorescence offers much faster analysis, high sensitivity, and enables a number of potential fluorescence-associated protein analysis applications such as capillary electrophoresis, fluorescence polarization, and fluorescence resonance energy transfer. 

## 2. Results and Discussion

### 2.1. Ca^2+^ Imaging of Ventricular Myocytes

**Figure 1:**
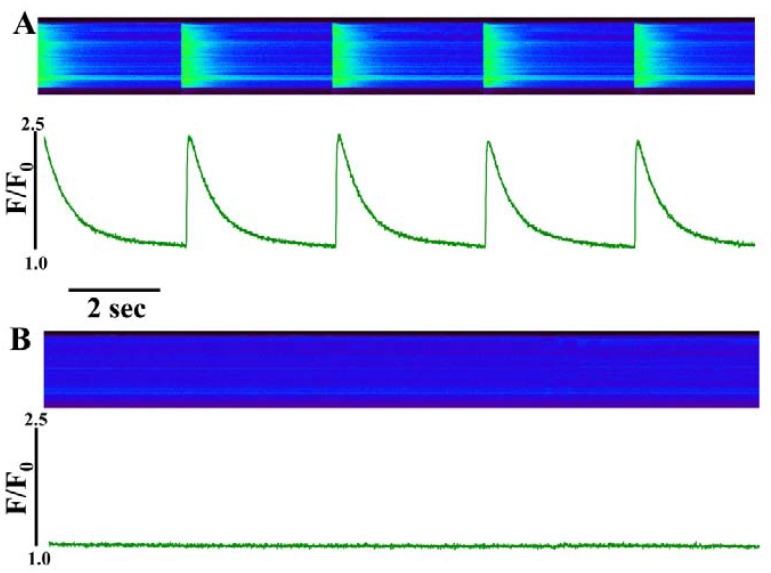
Perfusion of intact ventricular cardiomyocytes with a high concentration (30 µM) of IpTxa causes immediate cessation of [Ca^2+^]_i_ transients. (A) line-scan image (top) and associated fluorescence plot (bottom) of a field-stimulated mouse ventricular cardiomyocyte loaded with the Ca^2+^ indicator Fluo-4 and perfused constantly with normal Tyrodes solution in the absence of IpTxa (control). (B) The same cell ~1 min after perfusion with the same Tyrodes solution, but containing 30 µM IpTxa. The protocol was repeated four times and the result below is representative of three experiments.

IpTxa’s ability to induce subconducting states in single channels and to enhance binding of [^3^H]ryanodine to SR vesicles distinguish it as a RyR agonist. Furthermore, IpTxa’s high sequence identity with MCa suggests that the toxin may also be a cell-penetrating peptide. We therefore designed experiments to both investigate the effects of IpTxa on calcium signaling in intact cardiomyocytes, and to demonstrate its cell-penetrating capabilities. Ventricular cardiomyocytes were enzymatically digested, loaded with the Ca^2+^ indicator Fluo-3, and placed into the perfusion chamber of a Zeiss confocal microscope (see Experimental Methods). Cells were field-stimulated under Normal Tyrode (NT) solution, and Ca^2+^ transients were recorded in line-scan mode (control). Perfusion was then changed to NT + IpTxa (in the range of 100 nM-30 µM), and transients were recorded in the presence of toxin (+IpTxa). We hypothesized that if IpTxa penetrates cardiomyocytes to reach its intracellular target, it would alter the shape of [Ca^2+^]_i_ transients elicited with field-stimulation. As expected, changes in [Ca^2+^]_i_ transients were evident within seconds of perfusion with the toxin. Perfusion of IpTxa at high concentrations (30 µM) resulted in the complete cessation of [Ca^2+^]_i_ transients (Figure 1). For this experiment, cells were paced at 0.5 Hz under NT solution (see Experimental Methods). Cells were scanned first without IpTxa (control transients, Figure 1A), and then IpTxa was perfused and a second scan was taken immediately afterward (Figure 1B). 

**Figure 2:**
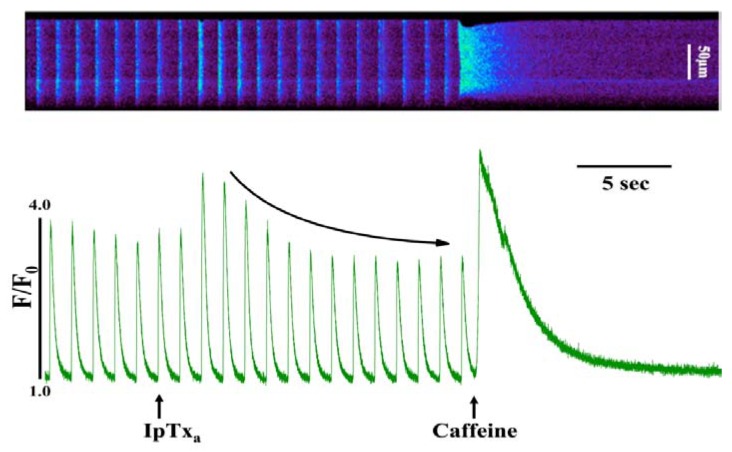
Perfusing intact cardiomyocytes with 100 nM IpTxa produced an increase in the amplitude of the [Ca^2+^]_i_ transient followed by a gradual decrease to a new steady-state. Line-scan image (top) and associated fluorescence plot (bottom) of a field-stimulated mouse ventricular cardiomyocyte loaded with the Ca^2+^ indicator Fluo-4 and perfused constantly with normal Tyrodes solution in the absence of IpTxa. At the time indicated by the arrow, IpTxa (100 nM) or caffeine (10 mM) was perfused onto the cell. The protocol was repeated 36 times and the result below is representative of 12 experiments.

At lower concentrations (100-300 nM), IpTxa perfusion elicited several discernible stimulatory effects in most cells. The most commonly observed response to perfusion with these IpTxa concentrations was a significant increase in the amplitude of the [Ca^2+^]_i_ transient, which was followed by a gradual diminution in transient amplitude until arriving at a new, lower steady-state (Figure 2). In similar [Ca^2+^]_i_ transient experiments, Eisner’s group found that 0.5 mM caffeine, a RyR agonist, elicited a brief increase in the amplitude of the Ca^2+^ transient, followed by a steady decline in amplitude to a new steady-state at a lower amplitude than in control transients [8]. They attributed the observed effects to partial depletion of SR Ca^2+^. The biphasic response elicited by IpTxa also closely mirrors that recently reported for Hadrucalcin, a recent addition to the calcin family, isolated from *Hadrurus gertschi* [9].

**Figure 3:**
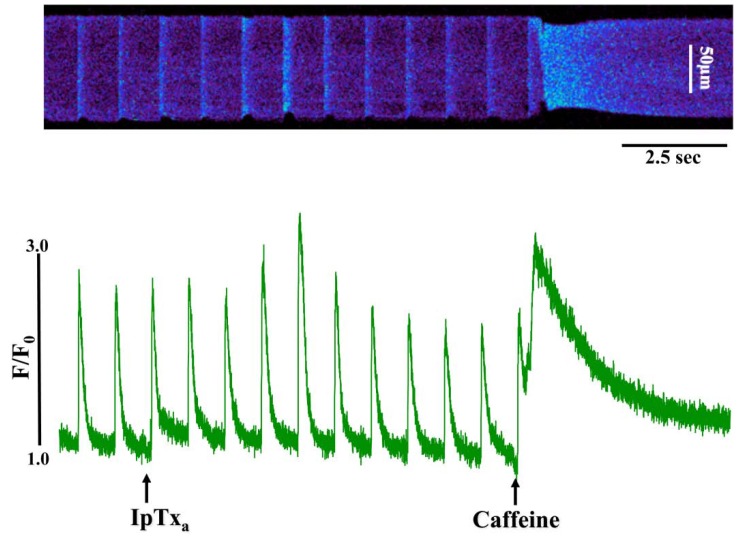
Perfusing 3 µM IpTxa increased diastolic [Ca]_i_ and produced irregular [Ca^2+^]_i_ transients. Line-scan image (top) and associated fluorescence plot (bottom) of a field-stimulated mouse ventricular cardiomyocyte loaded with the Ca^2+^ indicator Fluo-4 and perfused constantly with normal Tyrodes solution in the absence of IpTxa. At the time indicated by the arrow, IpTxa (3 μM) or caffeine (10 mM) was perfused onto the cell. The protocol was repeated 36 times and the result below is representative of three experiments.

In addition to the above response, we observed elevated diastolic [Ca^2+^]_i_ in some cells perfused with 100 nM to 3 µM IpTxa (Figure 3). This effect could be indicative of Ca^2+^ leak through RyRs, a possibility that we are exploring in a separate study. Finally, at all concentrations of IpTxa tested, cells frequently exhibited contractions that were discordant with field stimulation. These unsolicited contractions presumably result from a critical amount of Ca^2+^ leak through IpTxa-activated RyRs that is capable of propagating to neighboring release sites and activate IpTxa-free RyRs. Any of these Ca^2+^ release waves or leaks could potentially be a substrate for arrhythmias due to activation of the electrogenic sodium-calcium exchanger (NCX), which is known to induce delayed afterdepolarizations (DADs). However, the exact mechanism by which IpTxa elicits cessation of contractions, subtle Ca^2+^ leak or discrete Ca^2+^ waves requires further investigation.

### 2.2 Modification of IpTxa

Until recently, it was presumed that peptide toxins were incapable of crossing the plasma membrane and therefore to not recognize intracellular targets. The rapid onset of noticeable effects after IpTxa perfusion on intact cardiomyocytes strongly suggests that IpTxa crosses the cell membrane to reach its intracellular target rather than mimicking a membrane-embedded receptor, as was previously suggested [10]. Calcins, like other cell-penetrating peptides, are extremely basic peptides, with a net charge of at least +7 at physiological pH, which suggests that they, too, could permeate membranes [11,12]. It is also known that highly basic peptides translocate to the cytosol by locally perturbing the integrity of the lipid bilayer [13]. Translocation across the membrane could logically be the result of the interaction of the positive charges of the basic residues with negatively-charged polar heads of fatty acids of the plasma membrane. Indeed, in a recent series of elegant papers, MCa has been shown to translocate across cell membranes in a variety of cell types by virtue of an interaction of heparin and heparin sulfate and by macropinocytosis [14,15]. A linear analogue of MCa was shown to retain its cell-penetrating capability, though its pharmacological activity on RyRs was abolished [3].

In order to visualize the translocation of IpTxa into the cytosol of intact cells, we synthesized a fluorescently-labeled IpTxa derivative. It has been reported that Lysine 22 (K22) together with other basic residues (R23, R24, R31 and R33) form a critical domain of IpTxa that acts as a putative binding site and interacts with a region of the RyR [7,10,16]. In order to produce fluorescent derivatives of IpTx–a with minimum alteration of conformational or biological properties, we used a cyclic imidoester, 2-iminothiolane, to introduce a unique sulfhydryl group at the N-terminus without disrupting the critical binding site of the toxin. This addition made it possible to introduce subsequent modifications with fluorescent maleimide compounds. We used pH 7.0 in all reactions in order to ensure modification of the N-terminal group rather than ε-amino of the lysine residues. The mono-derivative was prepared using the labeling procedure described above. We then separated mono-2IT-IpTxa from unmodified toxin with HPLC and verified it with mass spectrometry (see Methods). 2IT-IpTxa was modified with fluorescent maleimide-Alexa-546, then separated and verified as above. The mono-modified derivative, Alexa-546-IpTxa, was used for subsequent chemical and biological characterization. 

### 2.3. Properties of Modified Imperatoxin A. Biological Activity and Fluorescent Properties

**Figure 4:**
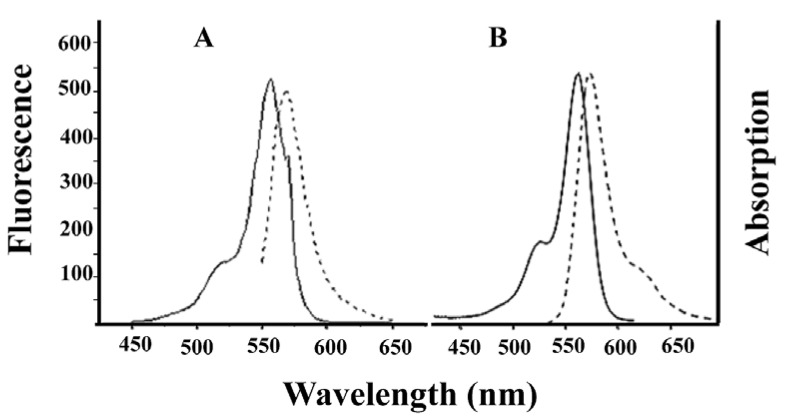
Absorption (solid line) and emission (broken line) spectra of Alexa-Fluor 546 (A) and Alexa-IpTxa (B). Unmodified IpTxa (not shown) lacks emission properties.

Alexa-Fluor-546-derivatized-IpTxa (Alexa-IpTxa) retained the spectral properties of the parent fluorophore (Figure 4). 

In addition, fluorescent IpTxa, like the parent molecule, enhanced the binding of [^3^H]ryanodine, although higher concentrations were needed to obtain the same half-maximal effective concentration (EC_50_). As reported previously, EC_50_ was 10 nM for native IpTxa, whereas Alexa-IpTxa exhibited an EC_50_ of 75 nM. Thus, modified IpTxa shows excellent activation of RyRs, which is only 7.5-fold lower than the native toxin. This result is impressive in light of previous chemical modification studies using scorpion toxins, which have indicated that modifications of amino groups lead to a loss of toxic activity [17]. This activity may be preserved, however, through guanidation of amino groups and limited iodination of tyrosine. These results suggest that the protonation state of the toxin is critical and that successful modification and introduction of fluorescent fluorophores depends on the preservation of the peptide’s net charge [21]. This improves the likelihood that local side chain interactions will be maintained, which in turn favors the conservation of the toxin’s tertiary structure and toxic activity.

**Figure 5:**
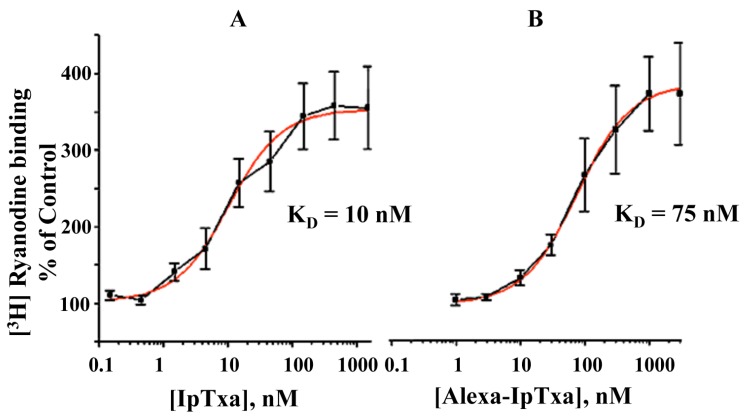
Dose-dependent activation of [^3^H]-ryanodine binding by native IpTxa and Alexa-IpTxa. Skeletal SR vesicles were incubated with 7 nM [^3^H]ryanodine in the absence (control 100%) and the indicated concentration of native (B) or modified IpTxa (A) The binding reaction was carried out for 90 min at 36˚C in medium containing 0.2 M KCl, 10 mM Na-HEPES (pH 7.2) and 10 μM CaCl_2_. Free and bound ligand were separated by rapid filtration as described in methods. Results are the mean ± S.D. of n=4 independent experiments.

### 2.4 Penetration of Intact Cardiomyocytes by Alexa-IpTxa

**Figure 6:**
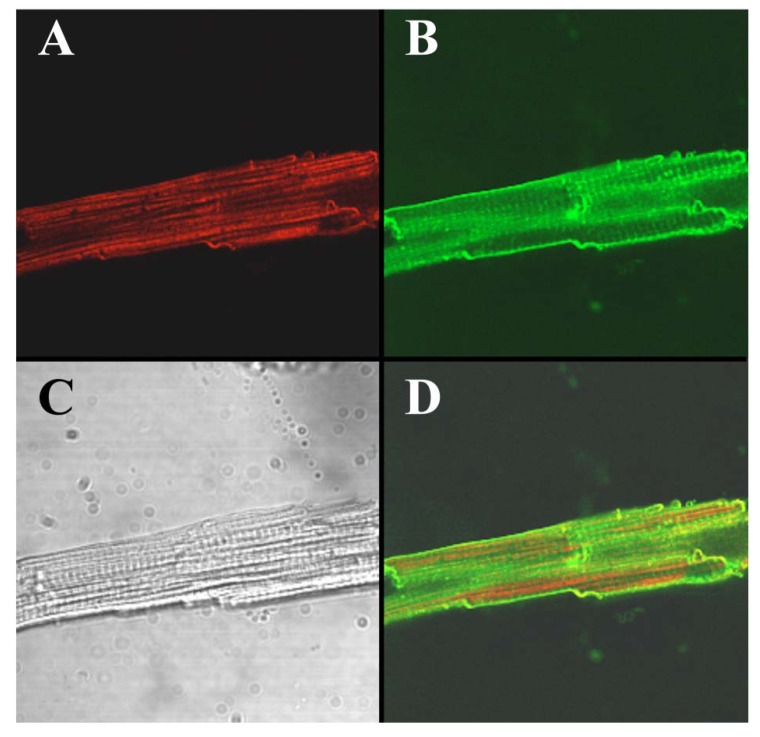
Alexa-IpTxa shows an intracellular localization in intact mouse ventricular myocytes. A, Alexa-IpTxa fluorescence; B, Alexa-Fluor-488 WGA; C, transmitted light image; D, merging of A and B images.

To visualize the cell-penetrating properties of Alexa-IpTxa in intact cells, ventricular cardiomyocytes were enzymatically isolated and incubated with 5 μM Alexa-IpTxa and Alexa 488-Wheat Germ Agglutinin (10 μg/mL) for 30 min at room temperature (see Experimental Methods). The distribution of Alexa-546-IpTxa (red fluorescence) and WAG-Alexa-488 (green fluorescence) were monitored in living, unfixed cardiac myocytes. Figure 6 shows images of a ventricular myocyte after this incubation. The transmitted light image is shown in C, Alexa-IpTxa staining in A, and wheat-germ agglutinin staining in B. Finally, D shows the merged image of A and B. It is clear that Alexa-IpTxa fluorescence localizes in the sarcoplasm of the cell, demonstrating that IpTxa, aside from being a potent and specific RyR activator, can cross the plasma membrane to interact with its target on the sarcoplasmic reticulum.

### 2.5 Kinetics of Cardiomyocyte Penetration by Alexa-IpTxa

**Figure 7:**
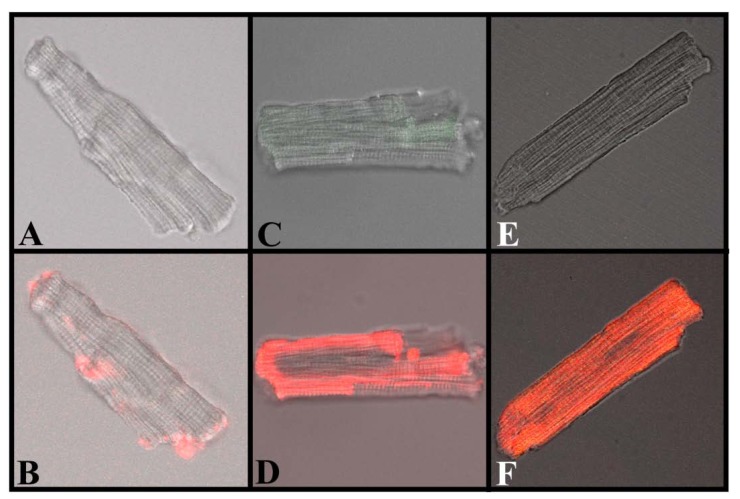
Translocation of IpTxa from external to intracellular compartments of intact mouse ventricular myocytes. All images represent the transmitted light image of the cardiomyocytes (C and B also show Fluo-3 fluorescence). A, C, and E were imaged at the beginning of the protocol, before the start of Alexa-IpTxa perfusion. B, Same cell as A, but after 10 minutes of Alexa-maleimide (1 μM) perfusion. D, Same cell as C, but after 10 minutes of Alexa-IpTxa (1 μM) perfusion. F, Same cell as E, but after 30 minutes of Alexa-IpTxa (1 μM) perfusion.

We next sought to establish a time-course for the translocation of Alexa-IpTxa across the plasma membrane of intact cardiomyocytes. Figure 7 shows transmitted light images of ventricular myocytes before (A, C, and E) and after incubation with Alexa-maleimide (B), which retains fluorescence but it is membrane-impermeable (thus serving as negative control for this experiment), or with Alexa-IpTxa (D and F), to verify penetration of the fluorescent derivative of IpTxa. Clear intracellular localization of fluorescence is seen only with Alexa-IpTxa, as early as 10 min after incubation (panel D), but more intensely after 20 min incubation (panel F). In order to refine the time-course of Alexa-IpTxa penetration into cardiomyocytes, we continuously monitored the evolution of fluorescence in 3 regions of interest (ROI) corresponding to the plasma membrane and adjacent cytosol (ROI-1), the bulk cytosol (ROI-2), and the nucleus (ROI-3). A fourth ROI (not shown) was defined to monitor changes in fluorescence of the bath solution. Animations of IpTxa translocation into cells are available online as supplementary data (URL: http://www.mdpi.com/1424-8247/3/4/1093/S1). 

**Figure 8:**
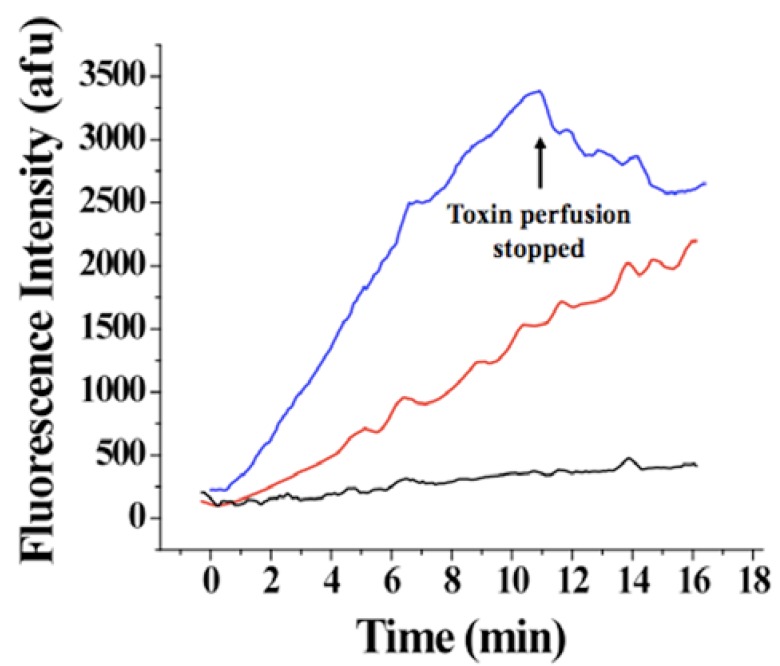
Evolution of Alexa-IpTxa fluorescence in three regions of interest. Blue, ROI-1, corresponding to the cell membrane and adjacent cytoplasm; Red, ROI-2 (bulk cytoplasm); Black, ROI-3 (nucleus).

An increase in fluorescence intensity was observed for ROI-1 and ROI-3 <50 seconds after the beginning of perfusion with 1 μM Alexa-IpTxa (Figure 8). A slower and much smaller increase in intensity was observed for ROI-3 (even after 30 minutes), suggesting that translocation across the plasma membrane into the cytosol (rather than plasma membrane to cytosol, then from cytosol to nucleus) is the more favorable pathway. The fact that fluorescence intensity was greater in all cellular compartments than that observed in the bath is indicative that intact cells act as toxin concentrators, as previously observed for MCa [7]. In contrast, cells perfused with Alexa-maleimide show only labeling of the membrane.

## 3. Experimental Methods

### 3.1. Reagents

[^3^H]Ryanodine was purchased from Dupont NEN (Wilmington, DE, USA). Phosphatidylethanolamine and phosphatidylserine were obtained from Avanti Polar Lipids, Inc. (Alabaster, AL, USA). Type II collagenase was acquired from Worthington Biochemical (Freehold, NJ, USA). Heparin was obtained from Wedgewood Pharmacy (Swedesboro, NJ, USA). Fluo-3 and Alexa-Fluor-546-maleimide was purchased from Invitrogen (Carlsbad, CA, USA). All other chemicals were purchased from Sigma-Aldrich, Inc. St. Louis, MO, USA). Indicator dye was first dissolved in dimethyl sulfoxide, then diluted further in buffer solution.

### 3.2. Preparation of Sarcoplasmic Reticulum Vesicles and [^3^H] Ryanodine Binding Assay

Heavy sarcoplasmic reticulum (SR) was prepared from rabbit white back and leg muscle using the procedure described by Meissner [18]. [^3^H]Ryanodine binding to rabbit skeletal SR was carried out as previously described [6,19]. Briefly, the standard incubation medium contained 0.2 M KCl, 1 mm Na_2_EGTA, 10 mM Na-Pipes pH 7.2, and CaCl_2_ to set free [Ca^2+^] to 10 μM. Ca^2+^/EGTA ratio was calculated using the stability constants of Fabiato [20]. [^3^H]ryanodine (68.4 Ci/mmol, Dupont NEN) was diluted directly in the incubation medium to a final concentration of 7 nM. Protein concentration was in the range of 0.2-0.4 mg/ml and was determined by Bradford method. Incubation lasted 90 min at 36°C. Samples (0.1 ml) were always run in duplicate, filtered on Whatman GF/C glass filters, and washed twice with 5 ml of distilled water using a Brandel M24-R cell harvester (Gaithersburg, MD, USA). Nonspecific binding was determined in the presence of 10 μM unlabeled ryanodine and has been subtracted from each sample. 

### 3.3. Ventricular Myocyte Isolation

Mouse ventricular myocytes were isolated by collagenase digestion of perfused mouse hearts using the method of Mitra and Morad [22] with modification addressed by Wolska and Solaro [23]. Mice were heparinized and anesthetized by cervical dislocation before removal and hanging of the heart. Cells were suspended in Tyrode solution ((mM) 130 NaCl, 5.4 KCl, 0.4 NaH_2_PO_4_, 0.5 MgCl_2_, 25 HEPES, 22 glucose, 0.01 μg/mL insulin, pH7.4 with NaOH) and kept at room temperature until used within 6 h. 

### 3.4. Ca^2+^ Imaging of Ventricular Myocytes

*Changes in cytosolic [Ca^2+^] using the Ca^2+^-dependent fluorescent dye Fluo-3 AM*. Fluo-3 AM was added to ventricular myocytes in Tyrodes solution (final concentration 10 μM) and incubated at room temperature for 30 minutes, after which the cells were washed and resuspended in Tyrodes solution. Fluorescence changes were monitored with a Zeiss META 5 line-scanning confocal microscope. Fluo-3 was excited at 488nm, and emissions were collected at 500-570 nm. Cells were perfused with normal Tyrodes and field-stimulated at 1 Hz. After recording control transients, synthetic, unmodified IpTxa was perfused at concentrations of 10 μM, 1 μM, and 100 nM for 10 seconds. To estimate the calcium load of the sarcoplasmic reticulum, we stopped stimulations and performed a rapid application of 10 mM caffeine to elicit complete Ca^2+^ release.

### 3.5. General Method for Chemical Modification of IpTxa with fluorescent fluorophores

Because native IpTxa does not contain free cysteine, new sulfhydryls were introduced through modification with the cyclic imidoester 2-iminothiolane (2IT), which preserves the net charge of the toxin. We added 54 nanomoles of 2IT to 100 μg (27 nmoles) of IpTxa in 50 mM potassium phosphate buffer (pH 7.0) plus 0.15 M KCl. The reaction mixture was incubated at room temperature for 18 h. The modification reaction was terminated by injection into a C_18_ column and eluted by HPLC with a gradient of acetonitrile (0 to 60% in 60min, solution A = 0.12% TFA in water, B = 0.1% TFA in 100% acetonitrile). The fraction that corresponded to IpTxa-2IT was indicated by mass spectrometry analysis. The sulfhydryl-containing toxin was then modified with fluorescent maleimide (Alexa-Fluor-546-C_5_-maleimide). Modification with Alexa was performed at room temperature for 6 h, using a 5-fold excess of maleimide dissolved in 50 mM potassium phosphate buffer (pH 7..0) plus 0.15 mM KCl. Fluorescence-labeled IpTxa was purified by gel filtration on a Bio-gel P4 column (1 × 30 cm) eluting with 50 mM phosphate buffer (pH 7.0) plus 1.0 M KCl. Fractions eluting at the void volume were subsequently purified by HPLC. The modified toxin was identified by mass spectrometry analysis.

### 3.6.Fluorescence Measurements

The fluorescence of Alexa-IpTxa was measured using a Hitachi Fluorometer F4500. The excitation wavelength was 570 nm, and the fluorescence emission spectra were collected at 450–600 nm with a peak at 550 nm. The emission wavelength was 510 nm and the excitation spectra were collected at 550-650 nm with a peak at 575 nm.

### 3.7. Confocal analysis of fluorescent IpTxa Derivatives

Isolated cardiac myocytes were incubated with Alexa-546-IpTxa (5 μM) with or without WGA-Alexa 488 (10 μg/mL) for 30 min in the dark. Images were acquired with a BioRad MR-1 confocal microscope coupled to a Nikon Disphot microscope and an X60 oil immersion objective using the Lasersharp 2000 software. Confocal images are 0.25 μm sections. All experiments were performed at room temperature. 

### 3.8. Timecourse for Alexa-IpTxa Transduction into Cardiomyocytes

Ventricular myocytes were imaged with a Zeiss META 5 confocal microscope equipped with laser scanning. Three regions of interest were delineated corresponding to the plasma membrane (ROI-1), the nucleus (ROI-2), and the cytosol (ROI-3). Changes in fluorescence intensity in these regions were monitored and analyzed. Alexa-546-IpTxa fluorescence was excited with the 488 nm line of an argon laser, and emitted light was filtered by a LP 585 filter. Alexa-546-IpTxa was perfused at a concentration of 1 μM. Scans were taken every 3 seconds for a total of 351 seconds. After the time series, perfusion was turned off and the cell remained in the bath for 30 minutes. Scans were taken at 9 minutes and 30 minutes. 

## 4. Conclusions

In this study, we have demonstrated that IpTxa, both in its native state and as a thiolated derivative, has the ability to cross the plasma membrane of intact cardiomyocytes to modulate its intracellular target, the sarcoplasmic reticulum Ca^2+^ release channel/ryanodine receptor (RyR). Penetration of native IpTxa into living, isolated cardiomyocytes is rapid (few seconds) and functionally consequential, as assessed by direct perfusion of IpTxa and visualization of [Ca^2+^]_i_ transients, whereas permeation of Alexa-IpTxa is slower (minutes), possibly due to the insertion of the membrane-impermeable fluorophore. The latter, however, shows that IpTxa is capable of delivering cargo to intracellular compartments, which opens an exciting field of intracellular drug delivery by RyR-targeting peptides. 

### 4.1. Effects of IpTxa on calcium signaling in intact cells

Perfusion of IpTxa on intact cells results in extremely rapid effects (lag time 2-3 sec) on intracellular Ca^2+^ signaling as evidenced by 1) an acute increase in the amplitude of the [Ca^2+^]_i _transient, indicative of enhanced Ca^2+^ release from the SR, followed by a gradual diminution in transient amplitude until reaching a new steady state at a lower amplitude than in control; 2) an elevation in diastolic [Ca^2+^]_i_ indicative of increased Ca^2+^ leak through RyRs; 3) cessation of [Ca^2+^]_i_ transients at high toxin concentrations; 4) unsolicited contractions. Except for 3, which is discussed below, these effects are similar to those observed after perfusion of hadrucalcin, a closely-related scorpion toxin [9], and to those of the RyR agonist caffeine. In the latter case, the alterations in Ca^2+^ signaling are attributed to depletion of Ca^2+^ from the SR [8]. However, complete cessation of Ca^2+^ transients would be incompatible with an *exclusive* effect of IpTxa on SR Ca depletion, inasmuch as Ca^2+^ entry via L-type Ca^2+^ channels is a small, but measurable component of the intracellular Ca^2+^ transient [4]. We do not have experimental support for the mechanism underlying the effect of high concentrations of IpTxa on Ca^2+^ transients, but we cannot rule out that high micromolar concentrations of IpTxa may totally abrogate Ca^2+^ transients by “upsetting” the external membrane and preventing membrane depolarization, thus exerting a less specific effect (or at least, one that would be independent of its action on RyRs). Although no such concentrations of IpTxa are ever reached in native environments and they rather represent experimenter-generated conditions, the effect warrants further investigation. 

The fact that IpTxa can acutely perturb Ca^2+^ signaling in ventricular myocytes holds promise for future investigation into diseases associated with dysfunctional Ca^2+^ handling such as catecholaminergic polymorphic ventricular tachycardia (CPVT), a genetic syndrome caused by dysfunctional Ca^2+^ release. CPVT results from various mutations of RyR2 that may go undetected until physical or emotional stressors augment pre-existing Ca^2+^ handling dysfunction, causing acute responses characterized by arrhythmias with the potential of degenerating into ventricular fibrillation. Though various animal models have been developed for the myriad of mutations associated with this disease, such models can be problematic due to compensatory Ca^2+^ removal mechanisms that could mask the mutant RyR phenotype. IpTxa, at low to moderate concentrations (as defined above), holds potential for a useful tool as a RyR modulator with the capability of rapidly inducing RyR hyperactivity unencumbered from effects on other channels or Ca^2+^ transport systems, allowing researchers to observe acute changes in Ca^2+ ^and subsequent compensatory changes as they occur in real time. Future research will be devoted to unraveling the mechanism by which IpTxa evokes these changes in Ca^2+^ signaling.

### 4.2. Alexa-IpTxa as a Fluorescent Cell-Penetrating Probe of RyRs.

In addition, we have described a method for the preparation of scorpion toxin derivatives containing fluorescent probes which are biologically active and retain high binding affinity for RyRs. The usefulness of these derivatives is two-fold, in that they can provide valuable information on the molecular environment of the toxin’s intracellular target and serve as a site-specific probe for RyRs. We have also demonstrated the capacity of IpTxa to permeate the external membrane, although the exact mechanism by which this translocation occurs remains to be explained. This characteristic makes IpTxa an excellent tool for the study of RyR in intact cells. Furthermore, the toxin’s ability to carry non-permeable cargo across the plasma membrane suggests the possibility that calcium channel-modifying drugs could be targeted specifically to RyRs, providing a novel therapeutic agent for the treatment of RyR channelopathies.

## Supplementary Files

Supplementary File 1 AVI-Document (AVI, 5536 KB)
